# Multidimensional Regulatory Mechanisms and Targeting Strategies of the eEF1 Family in RNA Virus Infection

**DOI:** 10.3390/v17050682

**Published:** 2025-05-07

**Authors:** Xin Wang, Kaituo Liu, Xiaoquan Wang, Xiufan Liu

**Affiliations:** 1 Key Laboratory of Avian Bioproducts Development, College of Veterinary Medicine, Yangzhou University, Yangzhou 225009, China; wangxin_yzu@163.com (X.W.); xfliu@yzu.edu.cn (X.L.); 2 Jiangsu Co-Innovation Center for Prevention and Control of Important Animal Infectious Diseases and Zoonosis, Yangzhou University, Yangzhou 225009, China; 3 Jiangsu Key Laboratory of Zoonosis, Yangzhou University, Yangzhou 225009, China; 4 Joint International Research Laboratory of Agriculture and Agri-Product Safety, The Ministry of Education of China, Yangzhou University, Yangzhou 225009, China

**Keywords:** eEF1 family, viral infection, translation elongation factor, host–virus interaction, antiviral targets, broad-spectrum antiviral drugs

## Abstract

The eukaryotic translation elongation factor 1 (eEF1) family exhibits critical roles in RNA viral infection beyond its canonical function in protein synthesis. This review analyzes the structural characteristics of eEF1A and the eEF1B complex, and their regulatory mechanisms during viral infection. eEF1A impacts viral replication by stabilizing viral RNA-dependent RNA polymerase (RdRp) complexes, modulating genomic RNA synthesis, and facilitating viral assembly through cytoskeletal regulation. eEF1B subunits contribute through enhancing viral mRNA translation, regulating nuclear transport of viral components, and mediating post-translational modifications. The high conservation of eEF1 proteins across species and their involvement in multiple stages of viral replication establish them as promising broad-spectrum antiviral targets. Current eEF1-targeting compounds like plitidepsin demonstrate efficacy against diverse viral families, though therapeutic development faces challenges in balancing antiviral activity with host toxicity. This review provides a theoretical foundation for developing novel antiviral strategies targeting host–virus interaction interfaces and offers insights into addressing emerging infectious diseases.

## 1. Introduction

Accelerated globalization and ecosystem changes have led to frequent outbreaks of emerging infectious diseases. When combined with the rapid mutability of viruses, traditional antiviral approaches targeting viral-specific components face significant challenges. Host cellular factors essential for viral infection are becoming a new direction for antiviral drug development due to their high evolutionary conservation across species and low susceptibility to mutagenesis. Among these, the eukaryotic translation elongation factor 1 (eEF1) family has demonstrated potential as a broad-spectrum antiviral target due to its extensive involvement in multiple processes of the viral life cycle.

Functionally, members of the eEF1 family not only regulate translational elongation through aminoacyl-tRNA delivery and GTP/GDP exchange [1,2,3,4], but also participate extensively in non-canonical processes including cytoskeletal regulation [5,6], nuclear export [7], apoptosis [8,9,10,11,12,13], autophagy [14], and others [15]. These functions are widely exploited by RNA viruses to enhance infection and evade immune defenses. Due to the evolutionary conservation of the eEF1 family and its regulatory roles in multiple stages of viral replication, targeting this family can achieve synergistic inhibition across different viral lineages while significantly delaying the development of drug resistance.

This review systematically analyzes the structural and functional diversity of the eEF1 family, elucidates its multidimensional regulatory mechanisms in RNA virus infection, and evaluates its translational potential along with existing challenges as an antiviral target. This work provides a theoretical foundation and innovative strategies for developing next-generation broad-spectrum therapies based on host–virus interaction interfaces.

## 2. Structural and Functional Architecture of the eEF1 Family

The eEF1 family, comprising eEF1A and the multisubunit eEF1B complex, serves as a central hub in cellular physiology through both canonical translation functions and non-canonical activities. The structural organization of these proteins, including their domains, isoforms, and quaternary arrangements, directly enables their functional versatility across protein synthesis, cytoskeletal dynamics, and cell survival pathways. This structural–functional relationship creates multiple interaction interfaces that viruses exploit during infection. This section examines the architectural features and interconnected activities of eEF1 family members that make them critical factors in both normal cellular processes and viral replication mechanisms.

### 2.1. Domain Organization and Isoform Diversity of eEF1A

**Figure 1:**
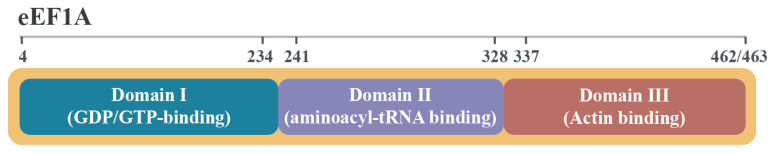
Domain structure and functions of eEF1A. eEF1A consists of three well-characterized structural domains: domain I (residues 4–234) is responsible for GTP/GDP binding; domain II (residues 241–328) participates in aminoacyl-tRNA binding; and domain III (residues 337–462/463) is involved in actin binding.

Eukaryotic elongation factor 1A (eEF1A) is one of the most abundant proteins in eukaryotic cells, exhibiting high evolutionary conservation in its molecular structure [6]. Structural biology studies have revealed that eEF1A possesses a typical three-domain organization: the N-terminal domain I, composed of approximately 230 amino acid residues, contains the GTP/GDP binding site and serves as the core region for its GTPase function; the central domain II is primarily responsible for recognizing and binding the aminoacyl end of aminoacyl-tRNA, ensuring protein translation fidelity through highly specific recognition mechanisms; the C-terminal domain III mainly mediates interactions with actin, playing an important role in the dynamic regulation of the cytoskeleton [5,16,17,18,19] (Figure 1). This precise modular spatial conformation enables eEF1A to form stable interaction networks with various molecular partners, thereby executing its diverse biological functions within the cell.

In mammals, eEF1A exists as two paralogous proteins, eEF1A1 and eEF1A2, which share 98% amino acid sequence similarity [20]. However, these two paralogous proteins exhibit significant differences in tissue distribution and function. eEF1A1 is widely expressed in most tissues, whereas eEF1A2 exhibits strict tissue specificity, with expression primarily restricted to neurons, skeletal muscle, and cardiac tissue [21]. This differential expression suggests the paralogous proteins’ distinct functions may link their tissue specificity to viral tropism and pathogenesis.

### 2.2. Subunit Composition and Assembly Mechanisms of the eEF1B Complex

The eEF1B complex, a crucial component of the eukaryotic translation elongation machinery, exhibits diverse subunit compositions and assembly mechanisms across species [22]. In yeast, eEF1B consists of two subunits: the catalytic guanine nucleotide exchange factor eEF1Bα and the structural protein eEF1Bγ [23]. Plants feature a trimeric complex comprising eEF1Bγ alongside two exchange subunits, eEF1Bα and eEF1Bβ [24]. Metazoans, however, form a more intricate heteromeric complex including eEF1Bγ, eEF1Bα, eEF1Bδ, and valyl-tRNA synthetase (Val-RS) [25].

The structural subunit eEF1Bγ, conserved across eukaryotes, contains glutathione S-transferase (GST)-like domains and serves as a scaffold, facilitating interactions with other subunits and subcellular localization [22,26,27,28,29,30]. eEF1Bα, universally present, harbors the catalytic exchange activity [31,32], while eEF1Bδ (in animals) and eEF1Bβ (in plants) represent lineage-specific exchange subunits with distinct regulatory roles. eEF1Bδ, unique to metazoans, possesses a leucine zipper (LZ) motif enabling dimerization and interaction with Val-RS, enhancing complex stability [33,34,35,36].

Currently, the nomenclature for eEF1B family factors is quite diverse; we have compiled the common naming methods in Table 1.

**Figure 2:**
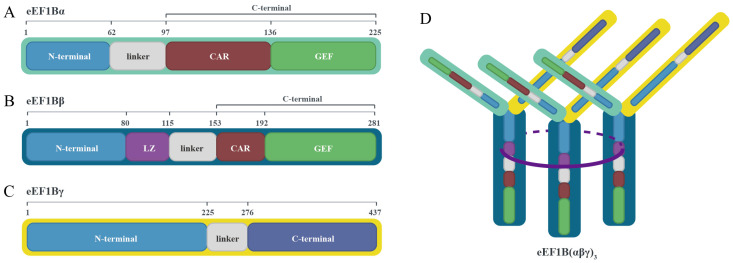
Structural model of the eEF1B(αβγ)₃ complex and domain organization of its subunits. (**A**) Domain architecture of eEF1Bα (light green): comprises an N-terminal domain, linker region, CAR, and GEF domain. eEF1Bα specifically interacts with eEF1Bβ via its N-terminal domain, while its GEF domain synergistically regulates complex function. (**B**) Domain architecture of eEF1Bβ (dark blue): contains an N-terminal domain, LZ motif, linker region, CAR, and GEF domain. eEF1Bβ forms a stable trimeric core via its LZ motif (helical bundle), with CAR and GEF domains extending outward from the core. (**C**) Domain architecture of eEF1Bγ (yellow): includes an N-terminal domain, linker region, and C-terminal domain. eEF1Bγ acts as a scaffold by simultaneously binding eEF1Bβ and eEF1Bα through its N-terminal domain. (**D**) Overall architecture of the eEF1B(αβγ)₃ complex: subunits α, β, and γ assemble in a 1:1:1 stoichiometry, forming a functional complex with six GEF domains. The LZ-mediated β-trimer forms the structural core, while α and γ stabilize the assembly via domain-specific interactions.

Recent structural studies have revealed a new trimeric model of the eEF1B complex: eEF1Bβ forms a stable trimer through its LZ motif. The C-terminus of each eEF1Bβ monomer contains a catalytically active GEF (guanine nucleotide exchange factor) domain and a central acidic region (CAR), which radiate outward from one side of the helical bundle core of the trimer (Figure 2B). eEF1Bα specifically interacts with eEF1Bβ through its N-terminal domain, while its own GEF domain synergistically participates in the functional regulation of the complex (Figure 2A). eEF1Bγ forms multiple interactions simultaneously with both eEF1Bβ and eEF1Bα through its N-terminal domain, primarily serving as a scaffold for complex assembly (Figure 2C). Ultimately, these three subunits bind in a 1:1:1 stoichiometric ratio, forming an eEF1B(αβγ)₃ complex containing six GEF domains [3,4] (Figure 2D).

### 2.3. Canonical Roles of the eEF1 Family in Protein Synthesis

The canonical roles of the eEF1 family in protein synthesis revolve around their essential functions during the elongation phase of translation, primarily involving the delivery of aminoacyl-tRNAs to the ribosome and the regeneration of GTP for continued elongation. During elongation, eEF1A in its GTP-bound form delivers aminoacyl-tRNA to the ribosomal A site [40]. Upon successful codon–anticodon pairing, eEF1A hydrolyzes GTP to GDP, subsequently releasing and departing from the ribosome [41]. eEF1A in its GDP-bound state requires reactivation before participating in the next round of translation elongation, which necessitates the involvement of the eEF1B complex [42].

**Figure 3:**
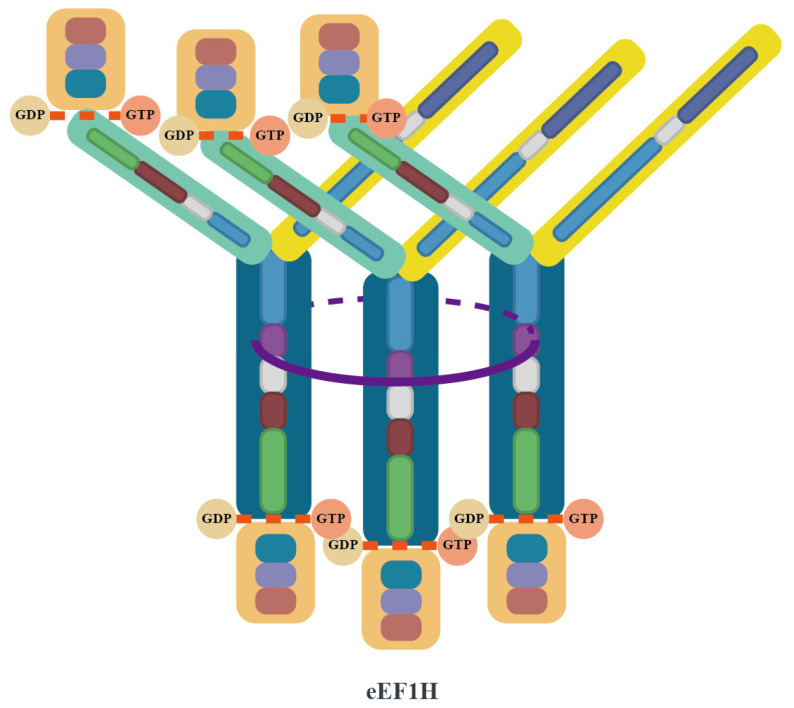
Structural architecture of the eEF1H supercomplex. The eEF1H supercomplex is organized around a trimeric core formed by three eEF1Bβ subunits (dark blue), which dimerize via their LZ motifs (purple) to create a stable helical bundle. Each eEF1Bβ subunit extends a GEF domain that directly binds an eEF1A molecule (orange), while its N-terminal domain interacts with an eEF1Bγ subunit (yellow). The eEF1Bγ subunits act as structural connectors, bridging the eEF1Bβ core to three eEF1Bα subunits (light green). Each eEF1Bα binds an additional eEF1A molecule through its GEF domain, resulting in a complete assembly of the eEF1B(αβγ)₃ complex (as detailed in Figure 2) integrated with six eEF1A molecules. The final architecture positions the six eEF1A molecules around the central β-trimer core, with the GEF domains of eEF1Bα and eEF1Bβ arranged to spatially coordinate their interactions with eEF1A.

The eEF1B complex catalyzes the conversion of eEF1A from its GDP-bound form to its GTP-bound form through its GEF activity, thereby reactivating eEF1A. Both the eEF1Bα and eEF1Bβ subunits within the complex contain GEF domains, which collectively form an efficient GEF hub capable of simultaneously binding up to 6 eEF1A molecules. This multivalent ligand binding characteristic and highly organized spatial arrangement may be a key mechanism for ensuring translation efficiency in higher eukaryotes. The supercomplex composed of eEF1A and eEF1B(αβγ)₃ is known as eEF1H [3,43,44] (Figure 3).

Additionally, the eEF1B complex further optimizes translation efficiency by binding to Val-RS, enabling direct transfer of valyl-tRNA and avoiding competition with other aminoacyl-tRNAs. This channeling mechanism is particularly important in higher eukaryotes as it compensates for the low affinity of valyl-tRNA for eEF1A [4].

### 2.4. Non-Canonical Functions of the eEF1 Family

#### 2.4.1. Cytoskeletal Dynamics

eEF1A participates in the construction and remodeling of the cytoskeleton through direct interactions with actin [17,45,46]. Research indicates that eEF1A can bind and bundle actin filaments, a function that does not depend on its translation elongation-related GTPase activity [47]. However, this function is competitively inhibited by aminoacyl-tRNA binding, suggesting a mutually exclusive regulation between these two functions [48]. In yeast models, overexpression of eEF1A or specific domain mutations (such as mutations in domains II and III that affect actin binding) results in actin cytoskeleton disruption [49,50]. The interaction with translation-related factors such as eEF1Bα can modulate eEF1A’s cytoskeletal function, revealing a coordinated regulatory network between translation and cytoskeleton dynamics [50].

The various subunits of the eEF1B complex influence cellular structure and function through interactions with different cytoskeletal components [6]. eEF1Bα directly binds to actin, regulating the balance between eEF1A’s roles in translation elongation and cytoskeletal organization [50,51]. eEF1Bγ interacts with keratin, participating in the bundling and positioning of intermediate filaments, thereby maintaining cellular mechanical strength and indirectly regulating protein synthesis efficiency [52,53]. Additionally, eEF1Bβ influences the dynamic reorganization of microtubule-associated structures during mitosis through phosphorylation-dependent changes in subcellular localization, potentially coordinating the spatial distribution of translation factors during the cell cycle [54,55]. These functions suggest that the eEF1B complex not only serves as an auxiliary factor in translation elongation but also participates in maintaining cell morphology and proliferation regulation by integrating cytoskeletal dynamics with translation mechanisms, thus demonstrating its multifunctionality in cellular physiological activities [56,57].

#### 2.4.2. Modulation of Apoptosis and Cell Survival Pathways

eEF1A dynamically regulates the balance between cell survival and death through isoform-specific mechanisms, with functions that are both isoform-selective and environment-dependent. While eEF1A1 typically exhibits pro-apoptotic characteristics during skeletal muscle differentiation [58], it demonstrates anti-apoptotic effects under specific pathological conditions such as ischemic brain injury. In these contexts, it interacts with HSC70 to inhibit the JNK signaling pathway, thereby blocking the cascade activation of Caspase 9 and Caspase 3 [12].

The neuron and muscle-specific isoform eEF1A2 maintains organelle function by activating autophagy to clear misfolded proteins [14], and can resist oxidative and endoplasmic reticulum stress by activating the AKT signaling pathway and inhibiting Caspase 3/8 activity [8,9]. Terminally differentiated cells (such as skeletal muscle and neurons) acquire resistance to apoptosis through developmental isoform expression switching (decreased eEF1A1 expression accompanied by increased eEF1A2 expression), which may be a key mechanism for homeostasis maintenance in long-lived cells [58,59]. However, tumor cells can abnormally exploit the pro-survival properties of eEF1A2; for example, by stabilizing MDM4 to suppress p53 function and promote liver cancer progression [60,61], indicating that its normal physiological functions can be hijacked under pathological conditions.

eEF1Bβ protects cells from stress-induced damage during heat shock response. Research shows that the long non-coding RNA (lncRNA) NONMMUT033452.2 can directly bind to eEF1Bβ and inhibit the expression of its downstream heat shock protein genes (such as Hsp60 and Hsp90). This inhibitory effect leads to elevated levels of reactive oxygen species (ROS) within cells while suppressing the proliferation of bronchial and alveolar epithelial cells, thereby exacerbating allergic airway inflammation [62,63].

## 3. Mechanistic Roles of the eEF1 Family in Viral Pathogenesis

The eEF1 family plays diverse roles during viral infection that extend beyond translation functions. These proteins interact with viral components at distinct stages of the viral life cycle, from genome replication to virion assembly and release. Despite their specificity, these interactions operate across multiple viral families, suggesting conserved mechanisms of host factor utilization. Notably, eEF1A and eEF1B subunits can either promote or inhibit viral replication depending on virus type and infection stage. This section examines the mechanisms and regulatory complexity of eEF1 family involvement in viral infection, highlighting the bidirectional nature of these interactions and their implications for viral pathogenesis and therapeutic development.

### 3.1. eEF1A in Viral Replication and Immune Evasion

#### 3.1.1. eEF1A-Driven Viral Genome Replication Mechanisms

Interaction with viral replicase enzymes is one of the core mechanisms by which eEF1A participates in RNA virus genome replication. During human immunodeficiency virus type 1 (HIV-1, family *Retroviridae*, genus *Lentivirus*, positive-sense RNA virus) reverse transcription, eEF1A interacts with reverse transcriptase (RT), providing essential support for viral DNA synthesis [64]. Similarly, research on tobacco mosaic virus (TMV, family *Virgaviridae*, genus *Tobamovirus*, positive-sense RNA virus) has shown that eEF1A binds to RNA-dependent RNA polymerase (RdRp) independently of viral RNA, and this interaction is critical in maintaining the catalytic activity of the polymerase [65]. The consistency of this direct enhancement of replicase function across diverse viral systems suggests that eEF1A likely serves as a common cofactor in viral replication machinery.

Beyond directly regulating replicase activity, eEF1A also promotes viral genome replication by participating in the assembly and stabilization of replication complexes. In respiratory syncytial virus (RSV, family *Pneumoviridae,* genus *Orthopneumovirus*, negative-sense RNA virus), eEF1A directly interacts with nucleoprotein (N protein) to stabilize the viral genome replication complex, thereby driving the genome replication process [66,67]. Tomato bushy stunt virus (TBSV, family *Tombusviridae*, genus *Tombusvirus*, positive-sense RNA virus) utilizes the specific binding of eEF1A to replication proteins p33 and p92^pol^, significantly enhancing the structural stability of the replication complex and consequently promoting efficient negative-strand RNA synthesis [68,69]. This complex stabilization mechanism also exists in prokaryotic systems, as seen with Qβ bacteriophage (family *Leviviridae*, genus *Levivirus*, positive-sense RNA virus), which employs the prokaryotic homologs of eEF1A, EF-Tu/EF-Ts, as essential cofactors for the RdRp complex, achieving enhanced replication efficiency by regulating the initiation phase of RNA synthesis [70].

The direct interaction between eEF1A and viral genomic RNA constitutes another important regulatory mechanism. Research on HIV-1 has demonstrated that eEF1A can directly bind to the 5’UTR region of the viral genomic RNA, a binding that is crucial for the late steps of reverse transcription, such as the synthesis of second-strand transfer DNA [71]. In West Nile virus (WNV, family *Flaviviridae,* genus *Orthoflavivirus,* positive-sense RNA virus) infection, eEF1A recognizes the conserved stem-loop structure at the 3’ end of the viral genome (3’-SL) to drive negative-strand RNA synthesis. Structural analyses reveal that this RNA interaction involves one primary binding site and two secondary sites, with disruption of any site leading to a drastic reduction in viral replication efficiency. Remarkably, while 3’-SL nucleotide sequences vary across *Orthoflavivirus* members (e.g., dengue virus (DENV), yellow fever virus (YFV)), their spatial conformation conservation enables eEF1A’s broad-spectrum binding—a refined evolutionary strategy for hijacking host factors [72,73]. This conservation highlights the potential for eEF1A to serve as a critical host node in arbovirus transmission cycles.

In the infection process of turnip yellow mosaic virus (TYMV, family *Tymoviridae*, genus *Tymovirus*, positive-sense RNA virus), eEF1A exhibits a more complex regulatory pattern. Studies have shown that eEF1A·GTP can bind to aminoacylated viral RNA and inhibit TYMV RdRp-mediated negative-strand RNA synthesis, while showing no such inhibitory effect on non-aminoacylated RNA [74,75,76]. Additionally, the tRNA-like structure (TLS) of viral RNA functions as an enhancer during translation, with its enhancing effect dependent on RNA aminoacylation and binding to eEF1A [77,78]. This mechanism likely coordinates the translation and replication functions of viral RNA during early infection, ensuring the orderly progression of the viral life cycle. These findings reveal that eEF1A not only promotes viral replication but also participates in precisely regulating critical transition points in the viral life cycle.

However, eEF1A’s regulation of viral replication is not unidirectionally promotive. In classical swine fever virus (CSFV, family *Flaviviridae*, genus *Pestivirus*, positive-sense RNA virus) infection, eEF1A interferes with replication complex assembly by binding to the NS5A protein and competitively occupying the viral internal ribosome entry site (IRES), inhibiting translation initiation efficiency [79]. In the avian influenza virus (family *Orthomyxoviridae*, genus *Influenzavirus A*, negative-sense RNA virus) (H5N1/HM) model, eEF1A mediates specific binding with PB1/PB2 subunits through the alanine residue at position 206, disrupting the stability of the PA–PB1 dimer, thereby inhibiting viral proliferation in A549 cells by regulating the assembly of the viral RNA polymerase complex (vRNP) [80]. This dynamic regulation exhibits phasic characteristics during the infection process: host cells resist viral invasion by upregulating eEF1A expression in the early stage of infection, while viruses break through host defenses by downregulating eEF1A expression in the later stage. This alternating interaction directly reflects evolutionary interplay at the molecular level.

#### 3.1.2. Viral Assembly and Release Mediated by eEF1A

When viruses enter the late stage of their life cycle, eEF1A shifts to participate in viral particle assembly and release processes. Studies have shown that eEF1A is widely present in purified particles of RNA viruses, including vesicular stomatitis virus (*Rhabdoviridae*) [81], severe acute respiratory syndrome coronavirus (SARS-CoV) (*Coronaviridae*) [82], and HIV-1 (*Retroviridae*) [83,84,85]. Notably, similar packaging has also been observed in selected DNA viruses such as vaccinia virus (*Poxviridae*) [86,87] and cytomegalovirus (*Herpesviridae*) [88,89], suggesting a conserved role of eEF1A in virion assembly across viral families.

In HIV-1, eEF1A interacts with the Gag polyprotein by binding to the matrix (MA) and nucleocapsid (NC) domains through its N-terminal 74-amino-acid region in an RNA-dependent manner. This interaction is mediated by basic residues within MA and NC, as mutations disrupting these regions (e.g., the AAA mutation in MA and the M1-2/BR mutation in NC) abrogate eEF1A binding. Experimental evidence demonstrates that a Gag double mutant (AAA M1-2/BR), which cannot interact with eEF1A, exhibits severe defects in viral particle assembly, underscoring the necessity of eEF1A for proper virion formation [83].

During the budding and release stage, eEF1A exerts its function through the regulation of cytoskeletal dynamics. In RSV infection, eEF1A promotes viral budding and release by regulating the dynamic changes of actin stress fibers. Inhibiting eEF1A function disrupts the formation of cellular stress fibers, impeding RSV-induced filopodia formation and significantly reducing viral release efficiency [66,67]. This intervention has minimal effect on RSV genome replication, indicating that eEF1A’s function in viral assembly and release is independent of its role in genome replication, highlighting the differentiated functions of eEF1A at different stages of the viral life cycle [67].

#### 3.1.3. Suppression of Host Immunity and Apoptotic Pathways

At the immunoregulatory level, eEF1A can influence host antiviral immune responses. Research by Gan et al. has shown that during SARS-CoV-2 (family *Coronaviridae*, genus *Betacoronavirus*, positive-sense RNA virus) infection, the viral protein NSP12 hijacks eEF1A to regulate the translation efficiency of host mRNAs, suppressing the production of type I interferons while promoting the expression of inflammatory factors. This facilitates viral replication and immune evasion, suggesting that eEF1A may promote viral replication by inhibiting host innate immune responses [90]. Additionally, eEF1A participates in the regulation of programmed cell death in infected cells by interacting with viral proteins (such as HIV Nef) to inhibit host cell apoptosis, thereby promoting viral survival [9]. These findings not only enrich our understanding of eEF1A functions but also provide a theoretical basis for developing novel antiviral strategies.

**Figure 4:**
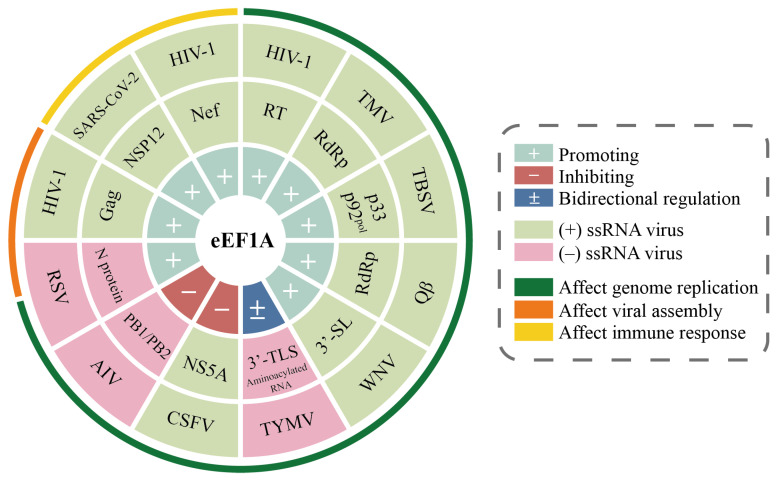
Hierarchical mapping of eEF1A−virus protein interactions and their regulatory roles across infection stages. The concentric circles (from inner to outer) illustrate: (1) regulatory effects of eEF1A on viruses (promoting or inhibiting viral infection); (2) viral proteins interacting with eEF1A; (3) names and types of viruses associated with eEF1A interactions; (4) stages of viral infection influenced by eEF1A.

The molecular mechanisms of eEF1A–virus interactions are systematically summarized in Table 2, while Figure 4 provides a hierarchical mapping of these interactions across infection stages.

### 3.2. eEF1B Subunits in Viral Lifecycle Regulation

#### 3.2.1. eEF1B2 Enhances Viral mRNA Translation Efficiency

eEF1B2 specifically binds to the 81–100 nucleotide region within the 5’ untranslated region (5’ UTR) of the Nipah virus (NiV, family *Paramyxoviridae*, genus *Henipavirus*, negative-sense RNA virus) M gene, significantly enhancing the translation efficiency of M mRNA. Deletion of nucleotides 81–100 markedly reduces both M protein expression and the release of virus-like particles (VLPs), demonstrating that the integrity of this region is essential for efficient M protein production. Given the critical role of the M protein in viral assembly and budding [92], this mechanism suggests that eEF1B2 may indirectly optimize viral replication by promoting M protein synthesis. However, the regulatory role of eEF1B2 on M protein translation was validated solely in a VLP model. Future studies using intact virus infections are required to confirm its direct contribution to viral budding [93].

#### 3.2.2. eEF1D Coordinates Nuclear Transport and Post-Translational Modifications

eEF1D counteracts influenza A virus (IAV, family *Orthomyxoviridae*, genus *Influenzavirus A*, negative-sense RNA virus) through multi-targeted interference with viral ribonucleoprotein (vRNP) dynamics. Gao et al. [94] demonstrated that eEF1D binds to all four subunits of IAV’s vRNP complex (PB1, PB2, PA, NP), with its RNA-dependent interaction with NP directly competing against importin α5 to suppress nuclear import of viral ribonucleoproteins. Concurrently, eEF1D weakens PB1–RanBP5 binding, thereby blocking nuclear entry of the PA-PB1 heterodimer and disrupting polymerase complex assembly. Furthermore, eEF1D inhibits NP oligomerization, a critical step for the formation of the PA-PB1-PB2 polymerase ternary complex, which ultimately destabilizes vRNP assembly. These coordinated actions collectively suppress both viral RNA (vRNA, cRNA, mRNA) synthesis and vRNP maturation, leading to a significant reduction in IAV replication efficiency.

Post-translational modifications also play a crucial role in eEF1D functional regulation. Studies have revealed that viral kinases (e.g., host cell cycle kinase cdc2 homologs) can target conserved sites on eEF1D, such as serine-133 (Ser-133), to regulate translation mechanisms [95,96]. For example, in herpesviruses (DNA viruses), kinases like herpes simplex virus type 1 (HSV-1) UL13 and Epstein–Barr virus (EBV) BGLF4 phosphorylate eEF1D at Ser-133, mimicking host kinase activity. Although the physiological role of this modification remains unclear, it suggests a broader viral strategy to hijack translation regulation that may extend to RNA viruses. Additionally, interactions between viral proteins (e.g., HSV-1 ICP0) and eEF1D further support the potential of eEF1D as a universal host target across diverse viral families [97].

#### 3.2.3. eEF1G Facilitates Viral Replication Complex Assembly

eEF1G forms functional interaction networks with IAV polymerase subunits (PB1, PB2, PA) and nucleocapsid protein (NP). Its knockout significantly inhibits the translation efficiency of viral structural proteins (such as M1, NP) but has no significant effect on the synthesis of viral RNA (vRNA/cRNA/mRNA) [98,99]. Notably, different strains of IAV exhibit significant differences in their dependence on eEF1G: the replication processes of A/WSN/33 (H1N1) and A/Perth/16/2009 (H3N2) heavily depend on the presence of eEF1G, while the 2009 pandemic A/California/04/2009 (H1N1pdm) virus strain shows independence from eEF1G. This difference suggests adaptive changes to host factors during viral evolution. Replacing the PB2 and PA of the WSN strain with those from the CA04 strain significantly reduces the recombinant virus’s dependence on eEF1G, suggesting that adaptive evolution of viral polymerase may achieve functional escape by reconstructing host factor interaction networks [98].

In the assembly stage of viral replication complexes, the non-structural protein 2B of foot-and-mouth disease virus (FMDV, family *Picornaviridae*, genus *Aphthovirus*, positive-sense RNA virus) can drive cellular membrane reorganization and vesicle formation by specifically binding to the C-terminal region of eEF1G (amino acids 208–437), thereby providing a structured platform for the assembly of viral replication complexes. Furthermore, this interaction may also promote the virus’s own transcription, localization, and translation processes, thereby enhancing viral replication capacity [100].

Members of the eukaryotic translation elongation factor family also demonstrate cooperative functions across viral families. For example, in TBSV infection, eEF1G and eEF1A cooperatively promote the synthesis of viral negative-strand RNA by binding to viral RNA and regulating RdRp activity, while simultaneously regulating the utilization efficiency of viral RNA templates, thereby driving efficient viral replication [101]. Similarly, in the HIV-1 infection system, eEF1G works synergistically with eEF1A on RT and integrase (IN), stabilizing the structure of the replication transcription complex (RTC) and ensuring the efficiency of the reverse transcription process [64,91]. The above studies indicate that eEF1 family members play crucial cooperative roles in the replication processes of different viruses and are important host factors for efficient viral replication.

**Figure 5:**
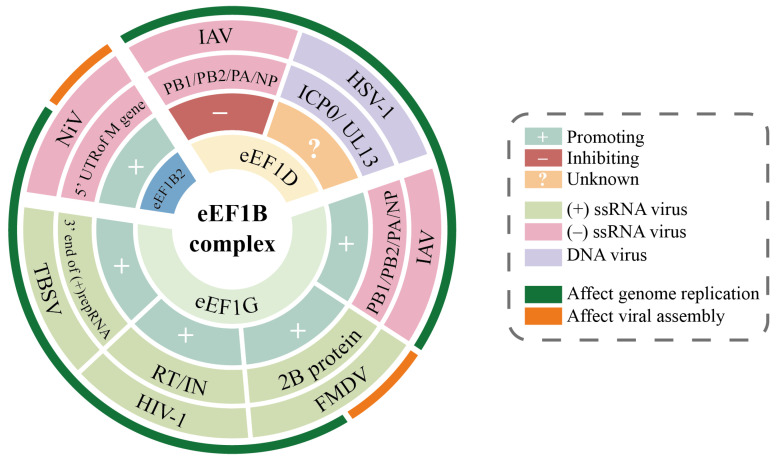
Viral proteins interacting with eEF1B and their regulatory effects. The concentric circles (from inner to outer) sequentially represent: (1) types of eEF1B subunits interacting with viruses; (2) regulatory effects of eEF1B on viruses; (3) viral proteins interacting with eEF1B; (4) names and types of viruses associated with eEF1B interactions; (5) stages of viral infection influenced by eEF1B.

The diverse roles of eEF1B subunits in viral infection are cataloged in Table 3. Figure 5 provides a complementary visualization of these interactions, mapping the specific viral proteins, infection stages, and regulatory outcomes associated with eEF1B subunits.

## 4. Strategies and Challenges in Developing eEF1-Targeted Antiviral Drugs

Recently, broad-spectrum antiviral strategies targeting host cellular factors have gained attention for their ability to circumvent drug resistance issues caused by viral genetic mutations. Among these targets, eEF1A has emerged as a promising candidate due to its critical role in viral replication cycles. Compounds such as plitidepsin, anisomycin, didemnin B, and oxazole-benzenesulfonamide derivatives exhibit cross-viral family activity through differential regulation of the eEF1A functional network. Their mechanisms include inhibition of viral protein synthesis and interference with reverse transcription processes. However, the off-target effects of these drugs on basic host physiological processes reveal a common dilemma of dose-limiting toxicity. Elucidating the mechanistic basis of both antiviral activity and host toxicity in eEF1A-targeting drugs could reconcile the critical dilemma between broad-spectrum efficacy and treatment safety in host-directed approaches. This would represent a pivotal advancement toward clinically viable next-generation antivirals.

**Figure 6:**
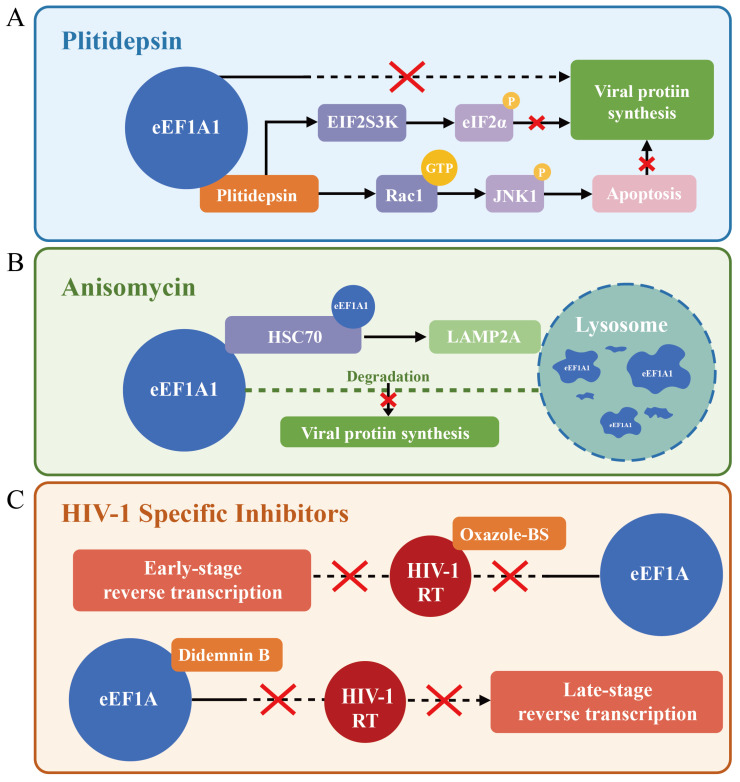
Mechanisms of antiviral drugs targeting the eEF1 family. (**A**) Plitidepsin inhibits viral protein synthesis by binding to eEF1A to block translation elongation, inducing eIF2α phosphorylation to disrupt translation initiation, and activating the Rac1–JNK1 pathway to trigger apoptosis, thereby enhancing antiviral efficacy. (**B**) Anisomycin inhibits viral replication by promoting the interaction between the host protein eEF1A and the chaperone HSC70, thereby inducing lysosomal degradation of eEF1A1 via the LAMP2A-dependent chaperone-mediated autophagy (CMA) pathway. (**C**) Oxazole-benzenesulfonamide derivatives interfere with the early-stage reverse transcription process by binding to HIV RT to block its interaction with eEF1A, without directly inhibiting RT enzymatic activity. Didemnin B suppresses the late-stage viral reverse transcription process by directly binding to eEF1A, disrupting its interaction with RT and destabilizing the RTC.

Plitidepsin achieves its antiviral effects through coordinated regulation of translation processes and stress response pathways. This drug specifically binds to the aminoacyl-tRNA binding domain of eEF1A, effectively blocking the delivery of aminoacyl-tRNA to the ribosomal A site during translation elongation [102]. Additionally, plitidepsin upregulates EIF2S3K kinase to induce eIF2α phosphorylation, interfering with the formation of translation initiation complexes [103,104,105], thereby effectively inhibiting viral protein synthesis at both the initiation and elongation stages. Beyond translational control, plitidepsin activates Rac1 GTPase, leading to increased intracellular ROS levels. This elevation in ROS promotes JNK1 phosphorylation and triggers cellular stress response pathways. Ultimately, these pathways initiate apoptotic programs, forming a secondary antiviral defense mechanism [106,107] (Figure 6A). This multi-target mechanism of action gives plitidepsin broad-spectrum activity across viral families, showing nanomolar inhibitory activity against *Coronaviridae* (including SARS-CoV-2 and its Omicron XBB1.5, BQ1.1 variants, MERS-CoV), *Flaviviridae* (HCV, ZIKV), *Herpesviridae* (HSV-1), and *Pneumoviridae* (RSV) [105,108].

In contrast to plitidepsin, anisomycin selectively degrades eEF1A1 through activation of chaperone-mediated autophagy, precisely blocking initial viral protein translation during early stages of enterovirus infection (Figure 6B). This mechanism not only inhibits the replication of coxsackievirus B3 (CVB3) but is equally effective against other enterovirus members such as enterovirus A71(EV71) and coxsackievirus A16(CVA16), suggesting the high conservation of eEF1A-dependent translation mechanisms across this viral family [109].

In the development of anti-HIV drugs, despite both didemnin B and oxazole-benzenesulfonamide derivatives targeting the RT–eEF1A interface, their mechanisms diverge by affecting different phases of reverse transcription. Didemnin B directly binds to eEF1A, disrupting its interaction with viral RT, which leads to decreased stability of the RTC, thereby inhibiting the late stage reverse transcription process [64,110]. In contrast, oxazole-benzenesulfonamide derivatives directly bind to RT, preventing RT–eEF1A interaction, interfering with early reverse transcription processes without directly inhibiting RT enzymatic activity [64]. Oxazole-benzenesulfonamide derivatives maintain activity against common NNRTI-resistant mutant strains (such as K103N and Y181C). This discovery provides an important theoretical foundation for developing novel anti-HIV drugs with potential to overcome resistance issues in existing medications (Figure 6C).

Despite the broad-spectrum antiviral potential of host-targeted drugs, their clinical translation still faces significant challenges. The core contradiction stems from eEF1A’s extensive involvement in basic cellular physiological processes, making these drugs prone to dose-dependent toxicity. Didemnin B has been withdrawn from clinical trials due to significant cytotoxicity caused by global interference with eEF1A function [110]. Oxazole-benzenesulfonamide derivatives’ side effects manifest as reduced cell viability and increased cell death. These compounds have minimal impact on cell viability at lower concentrations but still exhibit notable cytotoxicity at higher concentrations [64]. Anisomycin’s interference with lysosomal function may affect long-term medication safety [109]. Plitidepsin’s side effects include myalgia, liver function abnormalities, hematological abnormalities, and gastrointestinal symptoms, which are also closely related to its mechanism of inhibiting eEF1A and the drug’s pharmacological properties [102,111,112,113]. However, combination therapy with dexamethasone can effectively reduce the incidence of liver enzyme abnormalities, optimizing clinical safety [102]. This phenomenon suggests that combination drug strategies can balance efficacy and safety to some extent; nevertheless, developing broad-spectrum, low-toxicity host-targeted therapies remains the fundamental approach to solving this problem.

## 5. Innovative Directions for eEF1-Targeted Antiviral Therapies

To address these challenges, future research requires dual breakthroughs in molecular mechanism elucidation and technological innovation. From a mechanistic perspective, unraveling the regulatory networks of host eEF1 family factors during viral-host interactions represents a critical priority. Cryo-EM analysis of eEF1 family complexes with various viral proteins could reveal specific binding pockets exploited during host factor hijacking. These structural insights would provide the foundation for designing small molecules that selectively block virus–host interactions while preserving essential physiological functions.

From a technological perspective, combination therapies have demonstrated distinct advantages. The plitidepsin–dexamethasone combination effectively fights viral infection while suppressing host inflammatory responses and reducing the incidence of liver enzyme abnormalities [102,114]. This suggests that systemic modulation of host defense networks may offer improved safety profiles compared to single-target interventions. Of particular interest, combining host-directed agents with direct-acting antivirals creates synergistic mechanisms that simultaneously delay drug resistance development and reduce individual drug dosage requirements. For example, the plitidepsin–remdesivir combination has shown mechanistic complementarity in SARS-CoV-2 treatment [107,108].

In the field of delivery technologies, smart delivery systems that exploit tissue-specific microenvironments could solve toxicity challenges. Designing nanocarriers targeted to viral infection sites would concentrate drugs in affected areas while reducing exposure to healthy tissues. This targeted delivery approach could significantly lower side effects associated with host-targeting therapies.

From a broader perspective, antiviral therapeutic approaches are shifting from viral eradication to regulation of host defense homeostasis. Ideal host-targeting strategies should not merely suppress viral replication but establish a dynamic balance between defensive mechanisms and physiological equilibrium. This requires researchers to adopt an ecological view of host–virus interactions, identifying both conserved host factors essential for viral survival and the adaptive restructuring of host defense networks during infection. Future studies using single-cell sequencing and dynamic proteomics could track the spatiotemporal evolution of eEF1 family functional networks, potentially revealing precise molecular events in both viral hijacking and host counterattacks. These insights would guide stage-specific therapeutic interventions that maintain antiviral efficacy while reducing disruption of basic physiological processes, ultimately overcoming the traditional trade-off between broad-spectrum activity and treatment safety.

## Figures and Tables

**Table 1:** Nomenclature of common eEF1B complex components.

Merrick’s Nomenclature [37]	Current Nomenclature [3,22,33,38,39]	Prior Nomenclature [24,25]
eEF1Bα	eEF1Bα/EEF1B2/eEF1B2	eEF1β’(plant)
		eEF1β (yeast and metazoan)
eEF1Bβ	eEF1Bβ (plant) eEF1Bδ (metazoan)/ EEF1D/eEF1D	eEF1β (plant)
		eEF1δ (metazoan)
eEF1Bγ	eEF1Bγ/EEF1G/eEF1G	eEF1γ (yeast, plant and metazoan)

**Table 2:** Molecular mechanisms of eEF1A−virus interactions during infection.

Virus	Effect Phase	Molecular Mechanism	References
TMV	Genome replication	Binds to RdRp, maintains polymerase catalytic activity	[65]
RSV	Genome replication; virus release	Directly interacts with nucleoprotein to stabilize replication complex; regulates actin stress fibers to promote viral budding and release	[66,67]
TBSV	Genome replication	Specifically binds to replication proteins p33 and p92^pol^, enhances stability of replication complexes, promotes efficient negative-strand RNA synthesis	[68,69]
Bacteriophage Qβ	Genome replication	Acts as an essential cofactor for phage RdRp complex, participates in the initiation phase of RNA synthesis	[70]
WNV	Genome replication	Specifically recognizes the 3’ terminal stem-loop structure (3’-SL) of viral genome to directly promote negative-strand RNA synthesis	[72,73]
TYMV	Genome replication	eEF1A·GTP binding to aminoacylated viral RNA inhibits negative-strand RNA synthesis; binding to 3’-TLS enhances translation efficiency	[74,75,76,77,78]
CSFV	Genome replication	Competitively occupies IRES to inhibit translation initiation efficiency; binds to NS5A protein interfering with replication complex assembly	[79]
AIV	Genome replication	Mediates binding to PB1/PB2 subunits via Ala206, disrupts PA–PB1 dimer stability, affects vRNP assembly	[80]
SARS-CoV-2	Immune suppression	NSP12 hijacks eEF1A to regulate host mRNA translation efficiency, inhibits type I interferon production, promotes inflammatory cytokine expression	[90]
HIV-1	Genome replication; assembly and release; immune suppression	Functionally binds to RT and 5’UTR, supporting viral reverse transcription; binds to Gag protein, enhancing viral particle formation efficiency; interacts with Nef protein inhibiting host cell apoptosis	[9,64,71,83,84,85,91]

**Table 3:** Molecular mechanisms of eEF1B−virus interactions during infection.

Subunit	Virus	Effect Phase	Molecular Mechanism	References
eEF1B2	NiV	Genome replication; VLP budding	Specifically binds to the nucleotides 81–100 region in the 5’UTR of M gene, enhances M protein mRNA translation efficiency and VLP budding	[93]
eEF1D	IAV	Genome replication	Interacts with IAV polymerase subunits (PB1, PB2, PA) and NP; inhibits NP interaction with importin α5 reducing nuclear import efficiency; weakens PB1 binding to RanBP5 hindering PA–PB1 heterodimer formation	[94]
eEF1D	HSV-1	Genome replication	Viral kinase UL13 specifically phosphorylates Ser133 of eEF1D; ICP0 protein interacts with eEF1D	[95,96,97]
eEF1G	IAV	Genome replication	Interacts with IAV polymerase subunits (PB1, PB2, PA) and NP; significantly enhances translation efficiency of viral structural proteins (M1, NP)	[98,99]
eEF1G	FMDV	Genome replication; assembly and release	Interacts with non-structural protein 2B through C-terminal region (aa 208–437), drives cell membrane reorganization and vesicle formation	[100]
eEF1G	HIV-1	Genome replication	Works synergistically with eEF1A on RT and IN, stabilizes the structure of RTC, ensures high efficiency of reverse transcription process	[64,91]
eEF1G	TBSV	Genome replication	Works synergistically with eEF1A by binding to viral RNA and regulating RdRp activity to jointly promote viral negative-strand RNA synthesis	[101]

## Data Availability

The data presented in this study are available on request from the corresponding author.
